# Degradation of Decabromodiphenyl Ether in an Aerobic Clay Slurry Microcosm Using a Novel Immobilization Technique

**DOI:** 10.3390/microorganisms10020402

**Published:** 2022-02-09

**Authors:** Jung-Shan Hsu, Ting-Yu Yu, Da-Jiun Wei, Wann-Neng Jane, Yi-Tang Chang

**Affiliations:** 1 Department of Microbiology, Soochow University, No.70 Linxi Rd., Shilin Dist., Taipei 11112, Taiwan; rosalind@uab.edu (J.-S.H.); chowder0506@gmail.com (T.-Y.Y.); g11111155@gmail.com (D.-J.W.); 2 Department of Pathology, University of Alabama at Birmingham, P210 West Pavilion 619 South 19th Street, Birmingham, AL 35233-7331, USA; 3 Academia Sinica, Institute of Plant and Microbial Biology, 128 Sec. 2 Academia Rd., Nankang, Taipei 11529, Taiwan; wnjane@gate.sinica.edu.tw

**Keywords:** chitosan immobilization, decabromodiphenyl ether, photolysis, photocatalysis, biodegradation

## Abstract

A novel chitosan immobilization technique that entraps photocatalyst and microbes was developed and applied to decompose decabromodiphenyl ether (BDE-209) in a clay slurry microcosm. The optimized conditions for immobilization were obtained by mixing 1.2% (*w*/*v*) chitosan dissolved in 1% (*v*/*v*) acetic acid with nano-TiO_2_ particles and the BDE-209-degrading bacterial mixed culture. This aqueous mixture was injected into 1% (*w*/*v*) water solution containing sodium tripolyphosphate to form spherical immobilized beads. The surface of the immobilized beads was reinforced by 0.25% (*v*/*v*) glutaraldehyde cross-linking. These beads had enough mechanical strength during BDE-209 degradation to maintain their shape in the system at a stirring rate of 200-rpm, while undergoing continuous 365 nm UVA irradiation. This novel TiO_2_-Yi-Li immobilized chitosan beads system allowed a successful simultaneous integration of photolysis, photocatalysis and biodegradation to remove BDE-209. The remaining percentage of BDE-209 was 41% after 70 days of degradation using this system. The dominant bacteria in the BDE-209-degrading bacterial mixed culture during remediation were *Chitinophaga* spp., *Methyloversatilis* spp., *Terrimonas* spp. and *Pseudomonas* spp. These bacteria tolerated the long-term UVA irradiation and high-level free radicals present, while utilizing BDE-209 as their primary carbon resource. This new method has great potential for the treatment of a range of pollutants.

## 1. Introduction

Microbial bioremediation is considered to be an effective and environmentally friendly method for treating many emerging contaminants (ECs) present in soil and aquatic systems [1]. Polybrominated diphenyl ethers (PBDEs) have been identified as a class of ECs of great concern since they began to be used and they are still being used as brominated flame retardants in many industrial products worldwide. This means that their congeners are widely distributed in many environments [2]. For example, the concentration of PBDEs has been shown to be up to 37 mg/kg in soils sampled close to a PBDEs manufacturing factory in Jiangsu Province, People’s Republic of China [3]. Furthermore, exposure to a high level of PBDEs has been shown to result in developmental neurotoxicity, reproductive system issues, liver carcinogenesis, and the chemicals can even be passed on to offspring via breast milk [4,5]. Therefore, the development of an efficient treatment system that is able to decrease the level of PBDEs in the environment is a critical issue. A previous study has demonstrated that various species of microbes, such as *Pseudomonas* spp., *Terrimonas* spp., *Sphingomonas* spp., *Lysinibacillus* spp., *Bacillus* spp. and *Stenotrophomonas* spp., have the ability to biodegrade PBDEs [6,7,8]. However, microbial bioremediation requires a longer treatment time compared with chemical oxidation remediation or a modified bioremediation process [9,10]. To improve the efficiency of PBDEs biodegradation, one possibility is to combine various eco-friendly physical/chemical/biological processes [10,11,12].

The photocatalyst titanium dioxide (TiO_2_) has been shown to have a strong ability to accelerate photodegradation of ECs, including BDE-209 (C_12_Br_10_O), in aquatic systems when accompanied by UV irradiation [13]. One of the major limitations that have been reported is the aggregation of TiO_2_ nanoparticles, which significantly reduces the effectiveness of the photocatalytic reaction [14]. Efficiencies of ECs removal are then restricted by the weaker photocatalytic reaction due to a low UV light irradiation energy density; this is particularly a problem in a complex environmental matrix such as a soil slurry system. Moreover, to separate nano sized TiO_2_ by simple filtration after treatment is challenging due to their very small size; this limits the ability to reuse these particles [14,15]. In recent years, various techniques have been developed for immobilizing TiO_2_ using organic or inorganic materials, and these have included silica gel, films, membranes, concrete, glass, alginate beads and chitosan beads. The use of an immobilization process before treating the ECs makes it a relatively easy process to recover the TiO_2_ nanoparticles once treatment is completed [16,17,18,19,20,21,22].

There is little information available on the immobilization of a photocatalyst and microbes together within a single matrix at the same time and then the use of these beads to treat ECs ex situ. In order to combine microbial bioremediation with photocatalytic degradation after immobilization, the microbes and the TiO_2_ need to be entrapped simultaneously. The aim of this study is to utilize the chitosan-entrapped immobilization technique to develop an efficient removal system for remediating BDE-209 in a soil slurry system by combining photolysis, photocatalytic reactions and microbial degradation. The characteristics of the TiO_2_-microbes immobilized chitosan beads were assessed in this study, including the optimal chemical composition of the immobilization procedure; the microscopic surface of the beads; bacterial viability of the beads; bacterial entrapment and releasing ability of the beads. By analyzing BDE-209 removal in this system and pinpointing the changes that occur within the bacterial community during remediation, it is possible to evaluate the usefulness of the TiO_2_-microbes immobilized chitosan bead system for the biodegradation of BDE-209 present in a soil slurry.

## 2. Materials and Methods

### 2.1. Chemicals, Soil and Microorganisms

High-molecular-weight Chitosan (Sigma-Aldrich^®^, Iceland) was selected as the immobilization matrix material. The TiO_2_-nanoparticles (P25) were manufactured by Uni Region Bio-Tech Inc., Taipei, Taiwan. The BDE-209 (Alfa Aesar, Karlsruhe, Germany) used in this study had a purity above 99%. All other chemicals, including acetic acid, sodium tripolyphosphate (Na_5_O_3_P_10_), methyl blue (MB), and others, were reagent grade with a purity of >99%. All organic liquids, for example glutaraldehyde, were of HPLC grade with a purity of >99.9%. The Milli-Q water (>18 MΩcm) used in this study was obtained from a Millipore water purification system.

Pure Ca-montmorillonite was used as the clay slurry microcosm in this study, and this was purchased from the Clay Minerals Society, Purdue University, Indiana, USA. This clay was used to eliminate the confounding influence of soil organic matter (SOM), which is present in surface soils, and to a much lesser extent in subsoils, on UV photolysis and biodegradation. The physical-chemical characteristics of the sorbents are shown in Table 1.

The isolations of the indigenous bacterial mixed cultures used in this study were from PBDEs-contaminated river sediment and were carried out by our laboratory was described in a previous study [6]. The ability to remove BDE-209 by the Yi-Li and Ta-An bacterial mixed cultures has been shown previously to result in about 60% biodegradation of BDE-209 at initial concentration 25 mg/kg over 6 months when BDE-209 was the sole carbon source in a soil slurry microcosm [23]. The two mixed cultures were used to provide the bacteria within the two types of novel TiO_2_-microbes immobilization beads. These two types of novel beads thus ought to be able to utilize PBDEs as a sole carbon source and the bacteria within them should also be capable of growing well under long-term UVA irradiation [6].

### 2.2. Experimental Procedure

#### 2.2.1. Preparation of the TiO_2_-Yi-Li Immobilized Chitosan Beads

The scheme used in this study to create the TiO_2_ immobilized chitosan beads is shown in Figure S1. In total, 40 mL of Yi-Li bacterial mixed culture was concentrated into 2 mL by centrifugation and then 1.mL was added to and mixed with the TiO_2_-chitosan suspension. The rest of the immobilization procedure was the same as the procedure used for creating the TiO_2_ immobilized chitosan beads. The whole procedure was carried out under aseptic conditions.

#### 2.2.2. Bacterial Viability Assay of TiO_2_-Yi-Li Immobilized Chitosan Beads

First, 20 mL Yi-Li bacterial mixed culture were aliquoted and centrifuged at 3000 rpm for 5 min to remove the soil from the original culture. The supernatant was then transferred to a new tube and centrifuged at 10,000 rpm for 10 min. The pellet was resuspended in 1 mL 0.85% NaCl and added to each reagent for the designated time period and under the designated conditions as shown in Table 2. 

After serial dilution of the samples, 100 μL of each dilution was spread onto: (1) R2A agar for enumeration of soil heterotrophic bacteria; (2) BDE-209-mineral salts basal agar (BDE209-MSA) for the enumeration of BDE-209-biodegrading bacteria. After incubation, the colonies were counted and the bacteria numbers in the original sample were calculated.

#### 2.2.3. Degradation of Aqueous MB by the Photocatalytic Activity of TiO_2_ Immobilized Chitosan Beads

The aqueous MB (initial concentration is set up at 20 mg/L) degradation experiment was set up in order to evaluate the photocatalytic activity of TiO_2_ immobilized chitosan beads. Table 3 shows the detailed design of the experimental conditions for MB degradation. The degradation of MB using 0.01%, 0.05% or 0.1% TiO_2_ immobilized chitosan beads under continuing 365 nm UVA irradiation for 24 h. The optimal proportion of 10% (*w*/*v*) TiO_2_ immobilized chitosan beads was added and were shaken at 100 rpm at room temperature under various experimental conditions.

#### 2.2.4. Bacterial Entrapment and Release Ability Assay of the TiO_2_-Yi-Li Immobilized Chitosan Beads

**Figure 1:**
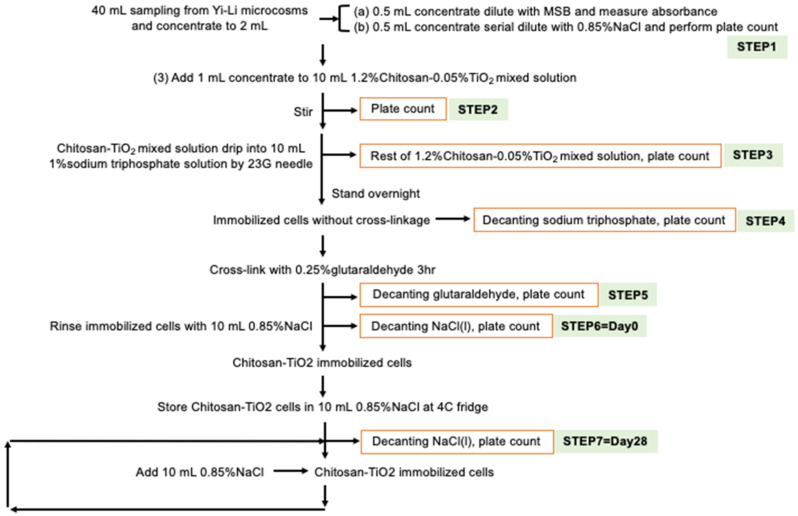
Immobilization Procedure used to Create the TiO_2_-Yi-Li Immobilized Beads.

The same protocol, as described in Section 2.2.2, was performed on the TiO_2_-Yi-Li immobilized chitosan beads. Specifically, 100 μL of solution from each step, as showed in Figure 1, was used and plated as described. After incubation, the colonies were counted and bacteria numbers in the original sample calculated.

#### 2.2.5. Degradation of BDE-209 in a Clay Slurry Microcosm by the TiO_2_-Yi-Li Immobilized Chitosan Beads

BDE-209-contaminated clay were initially prepared at 20 mg/kg, as described in a previous study [24]. Batch biodegradation experiments used the BDE-209 (20 mg/kg) as the sole carbon source and were performed in an aerobic clay slurry microcosm system. The ratio of clay to mineral salts basal medium (MSB) was 2:25 (g/mL). The clay slurries were sterilized, and this was confirmed by spread plates. At the beginning of the experiments, the optimal proportion of 10% (*w*/*v*) TiO_2_-Yi-Li immobilized chitosan beads was added to the clay slurry microcosms (data not shown). The clay slurry microcosms were incubated with shaking at 100 rpm at room temperature under various experimental conditions. These were: (1) biodegradation alone by TiO_2_-Yi-Li bacteria immobilized cells; (2) photodegradation alone by the 365 nm LED UVA irradiation at 2.0–2.4 mW/cm^2^; (3) both biodegradation and photodegradation; (4) the negative control microcosms were sterilized with the biocide NaN_3_ (ca 1.0%, *w*/*v*). Samples for the experimental analysis were taken from each individual microcosm.

### 2.3. Analytical Methods

#### 2.3.1. Analyses with a Scanning Electron Microscope

A cryogenic scanning electron microscope (SEM, FEI Quanta 200/Quorum PP2000TR FEI, Plant Cell Biology Core Laboratory, The Institute of Plant and Microbial Biology, Academia Sinica, Taiwan) was used to carry out the observations of the immobilized cell samples at 5 kV. The beads containing the immobilized cells were initially cut by scalpel and then they were fixed on a specimens-stage using TedPella PELCO^®^ conductive graphite gel. Next the specimens were pretreated with a sequence of freeze-dried and Au sputtering processes. Following this procedure, the specimens were frozen using a manifold freeze dryer and then are dried at −80 ℃ overnight. Finally, energy dispersive X-ray (EDX) analysis on immobilized cell samples was performed by the field emission-SEM using a EDX spectrometer (Hitachi S-4700 type-II with Horiba EMAX-ENERGY EX-300, National Tsing Hua University, Hsinchu, Taiwan).

#### 2.3.2. BDE-209 Analysis

A serial process involving ultrasound-assisted extraction, solid-phase extraction, and vacuum concentration was carried out on the clay slurry microcosm samples. After this pretreatment, the samples underwent HPLC analysis to detect and measure BDE-209. The HPLC system was equipped with a UV detector (YL-9100, Young-Lin, South Korea) at 226 nm, an ODS Hypersil C18 column (250 mm × 4.6 mm, Thermo Scientific, Waltham, MA, USA) and was operated at 30 ℃. The operating conditions of the HPLC system were as follows: 25 μL injection sample and 1 mL/min mobile phase 100% acetonitrile. Triplicate analysis of the BDE-209 in each sample was carried out. The retention time of BDE-209 on this HPLC chromatographic system was 8.916 min.

#### 2.3.3. Bromide Analysis

Bromide (Br^−^) byproduct concentrations were measured at intervals during debromination as the BDE-209 degradation in a clay slurry microcosm by TiO_2_-Yi-Li immobilized beads took place. This was carried out using an ion chromatograph (IC, Metrohm 883/863, Switzerland) equipped with a conductivity detector. Samples of the supernatant liquid were separated by high-speed centrifugation, filtered through a 0.22-μm nylon filter, and then their Br^−^ concentration was measured immediately. The retention time for Br^−^ by IC chromatography was 8.29 min.

#### 2.3.4. Bacterial Count Analysis

The bacteria were incubated on R2A agar (HIMEDA^®^, Mumbai, India) to count the total number of heterotrophic bacteria able to grow on this medium. Restrictive BDE209-MSA agar was also used to count the bacteria identified in this system. Each sample was diluted 10^1^–10^10^-fold using sterilized saline solution (KH_2_PO_4_ and MgCl_2_ 6H_2_O) after the sample had been collected; then, 0.2 mL of each dilution was spread on the relevant agar plates and incubated at room temperature for up to 48 h.

#### 2.3.5. Bacterial Community Analysis

Bacterial community analysis was applied to assess the changes in the bacterial community during the BDE-209 degradation. At two time points, day 0 (D0) and day 56 (D56). DNA samples were extracted using a Soil Genomic DNA Purification Kit (GeneMark, Taipei, Taiwan). The bacterial communities were then analyzed using the MiSeq, paired-end 300 bp primer set [25], which amplifies the V3–V4 domains of 16S rDNA of the bacteria on an Illumina 454 (Biotools, Taipei, Taiwan). The DNA sequences present in the amplified samples were processed into an amplicon library and this was followed by sequencing using synthesis sequencing. Finally, after the results were subjected to cluster generation, the bacterial communities present in the samples during the BDE-209 biodegradation were investigated.

#### 2.3.6. MB Analysis

Absorbance measurements were carried out on a spectrophotometer and degradation of MB at a wavelength of 664 nm was measured.

## 3. Results

### 3.1. Characteristics of the TiO_2_-Yi-Li Immobilized Chitosan Beads

#### 3.1.1. Determination of the Chemical Concentrations to Be Used in the Immobilization Procedure

In order to determine the optimal concentrations of chitosan, sodium tripolyphosphate, and glutaraldehyde to be used in the immobilization procedure, preliminary tests were carried out under the conditions shown in Table 4.

Six batches of TiO_2_ immobilized chitosan beads were made using different concentrations of reagents. The total weight and the appearance of the TiO_2_ immobilized chitosan beads made from 50 mL of chitosan-TiO_2_ mixed suspension were the two factors used to determine the optimal concentrations of each reagent needed for the immobilization procedure. Previous studies suggested that the concentration of chitosan in the solution should be in the range between 1.2% to 4.5% and that gelling would require the addition of an aqueous solution of tripolyphosphate at a concentration within the range between 1% and 3%; finally, to reinforce the surface of beads, glutaraldehyde at a concentration of 0.25% would be needed in order to obtain regular shaped beads that have good protection against environmental shear forces [26,27,28]. Batches one, two and three failed to form regular shaped beads, batches four and five did result in enough spherical beads, but the diameters of the beads were not consistent. In the end, batch six gave the best TiO_2_ immobilized chitosan beads and the condition used for this batch were: 1.2% (*w*/*v*) chitosan mixed with TiO_2_ nanoparticles dissolved in 50 mL 1% (*v*/*v*) acetic acid and gelling within 1% (*w*/*v*) sodium tripolyphosphate. The surface of beads was reinforced by soaking in 0.25% glutaraldehyde (*v*/*v*). In total, 12.5 g TiO_2_ of immobilized chitosan beads were able to be produced, and these had an average diameter of 2 mm.

#### 3.1.2. Viability of the BDE-209-Degrading Microorganisms That Have Been Immobilized in the Chitosan Beads

**Figure 2:**
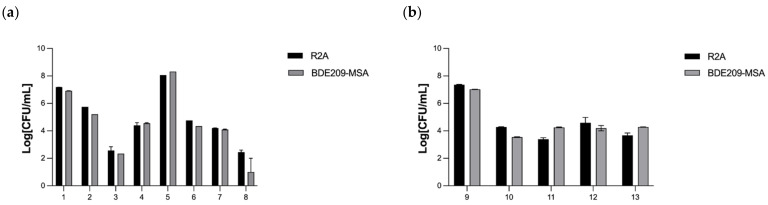
The viability of the Yi-Li bacteria in various chemicals used during the immobilization procedure. (**a**) Yi-Li bacteria viability (**b**) Yi-Li bacteria viability under UV irradiation. Value 1–13 on the X axis is defined as each step of Table 2, including the various chemicals used during the immobilization procedure. Step: 1: Control-0.85% NaCl; step 2: 1% acetic acid; step 3: 0.1% TiO_2_ in 1% acetic acid; step 4: 0.05% TiO_2_ in 1% acetic acid; step 5: 1% sodium triphosphate; step 6: 0.25% glutaraldehyde; step 7: 1.2% chitosan dissolved in 1% acetic acid; step 8: 1.2% chitosan dissolved in 1% acetic acid; step 9: Control-0.85% NaCl; step 10: 1% acetic acid; step 11: Chitosan-0.1% TiO_2_ mixed suspension; step 12: Chitosan-0.05% TiO_2_ mixed suspension; step 13: Chitosan-0.01% TiO_2_ mixed suspension.

The bacteria viability test was set up to confirm that the BDE-209-degrading bacterial mixed culture is able to survive in contact with the various reagents used in immobilization process before degradation of BDE-209 by TiO_2_-Yi-Li immobilized chitosan beads in an aerobic clay slurry microcosm; this is because chitosan, glutaraldehyde and TiO_2_ have all been reported to have antibacterial ability [24,29,30]. In this set of tests, two different BDE-209-degrading bacterial mixed cultures were used, Yi-Li and Da-An, to create the beads [6]. The results of the Yi-LI bacteria viability test are presented in Figure 2. 

The number of viable Yi-Li bacteria were found to decrease when they were in contact with chitosan, TiO_2_ and glutaraldehyde. Nonetheless, the Yi-Li bacteria were able to survive all of the chemical reagent steps used in the immobilization procedure and seem to be quite resistant to the level of free radicals released by the TiO_2_ under continuous 365 nm UVA irradiation. As Figure S2 shows, the Da-An bacteria were killed by 0.25% glutaraldehyde and the viable Da-An bacteria were not present beyond this point. Therefore, the Yi-Li bacteria, and not the Da-An bacteria, was selected to be immobilized in the chitosan beads for the remaining experiments.

**Figure 3:**
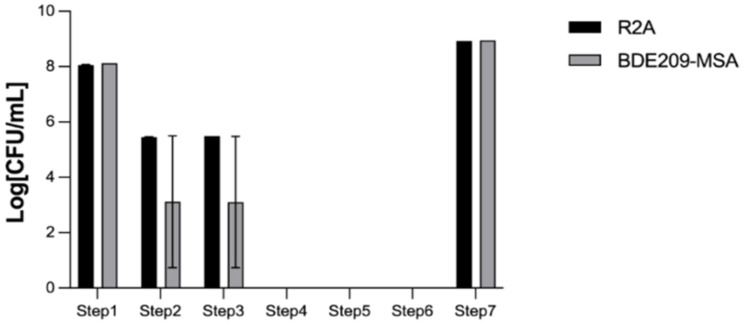
The bacterial entrapment and release ability of TiO_2_-Yi-Li immobilized chitosan beads. Value on the X axis is defined as each step of Table 3.

The bacterial entrapment and releasing ability of the TiO_2_-Yi-Li immobilized beads are shown in Figure 3. 

The purpose of this experiment was to indirectly confirm that the immobilization technique was able to entrap the bacteria within the chitosan beads and then slowly release the bacteria from the immobilized beads into the environment. Step 1 found that the initial bacterial number added to the TiO_2_-chitosan mixed suspension was 1.31 × 10^8^ CFU/mL. Step 2 shows the remaining bacteria after mixing the bacteria with the TiO_2_-chitosan mixed suspension. Step 3 shows the number of remaining bacteria, 2.96 × 10^5^ CFU/mL, after contact for 30 min with the TiO_2_-chitosan mixed suspension. The bacteria numbers slightly dropped during Step 2 and Step 3 since both chitosan and TiO_2_ have an antibacterial effect. At Step 4, there was no bacteria present in the decanted sodium tripolyphosphate solution, which indicates that all of the added bacteria were entrapped within the chitosan immobilized beads. At Steps 5 and 6, no bacterial were detected in the decanted glutaraldehyde and NaCl, which confirms that the bacteria were still immobilized within the chitosan beads without leakage. At Step 7, which was carried out 28 days after beads had been made, the number of bacteria released into aqueous solution was estimated to be 8.9 × 10^8^ CFU/mL, which is more than the initial number of Yi-Li bacteria added during the immobilization process. The bacterial release profile was carried out in an aqueous solution; yet, bacteria entrapped in the beads, once released, can recover and thrive under such conditions, although no nutrients are available in the aqueous solution. Thus, it would seem that the Yi-Li bacteria were able to recover from the immobilization process, divide and finally become released from the chitosan immobilized beads into the outside environment.

#### 3.1.3. The SEM Microphotographs and EDX Profile of the TiO_2_ Immobilized Chitosan Beads

**Figure 4:**
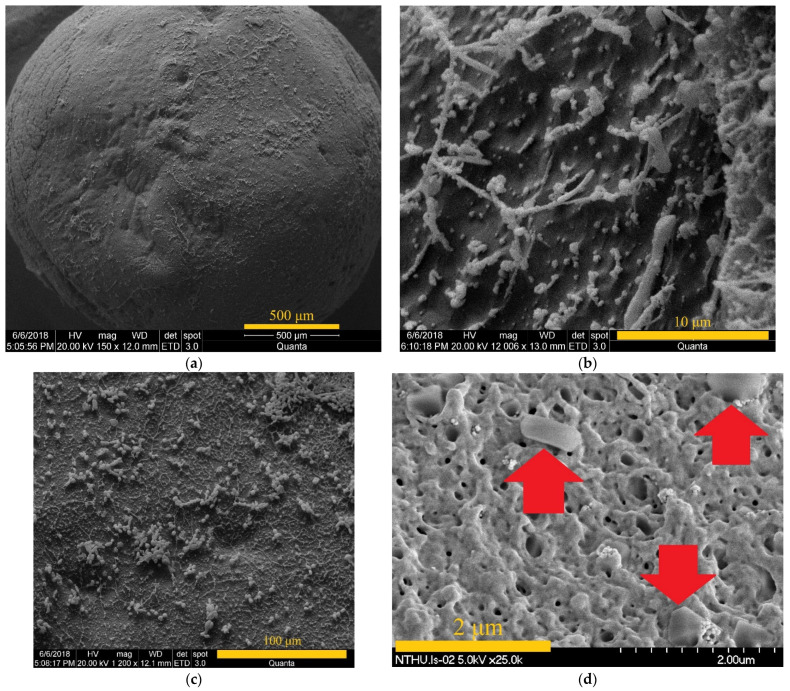
SEM microphotographs of the surface of the beads. They are magnified by (**a**) ×150 and the cross-section magnified by (**b**) ×1200 of TiO_2_ immobilized chitosan beads and **(c**) ×12,006 and (**d**) ×25,000. Length scale consist of an orange bar. Bacteria marked by red arrows.

**Figure 5:**
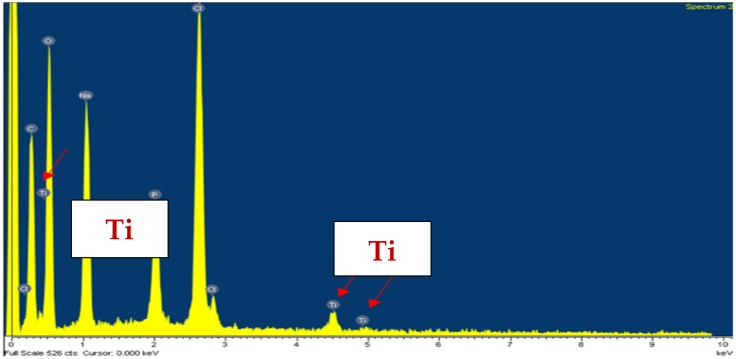
EDX analysis of the TiO_2_-Yi-Li immobilized chitosan beads. Signs of Ti marked by red arrows.

The SEM microphotographs of the TiO_2_ immobilized chitosan beads and the TiO_2_-Yi-Li immobilized chitosan beads are shown in Figure 4. EDX analysis of the TiO_2_-Yi-Li immobilized chitosan beads is shown in Figure 5. The aggregated TiO_2_ can be observed in Figure 4 on the surface of the bead (a), (b), and (c) at different magnifications. The rod-shaped or circle shaped subject labeled in Figure 4. (d) with red arrow would seem to be a bacillus or coccoid form bacterium. 

### 3.2. Degradation of BDE-209 by TiO_2_-Yi-Li Immobilized Chitosan Beads in an Aerobic Clay Slurry Microcosm

#### 3.2.1. BDE-209 Degradation

**Figure 6:**
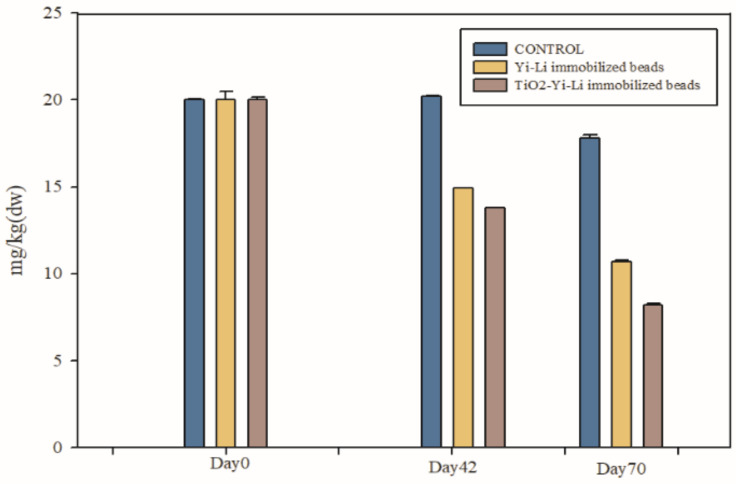
BDE-209 degradation by TiO_2_-Yi-Li immobilized chitosan beads and Yi-Li immobilized chitosan beads.

To confirm ability of the TiO_2_-Yi-Li immobilized chitosan beads to remove BDE-209 in a clay slurry microcosm and also to determine the contribution to this process of the Yi-Li bacteria and the TiO_2_, a clay slurry microcosm containing BDE-209 was set up. The TiO_2_-Yi-Li immobilized chitosan beads or Yi-Li immobilized chitosan beads were added the slurry systems and UVA irradiation begun; this time point was recorded as Day 0 (D0). The concentration of BDE-209 remaining in the clay slurry on Day 0 (D0), Day 42 (D42) and Day 70 (D70) were measured and are shown in Figure 6. 

The initial concentration of BDE-209 for both systems on D0 were 20 mg/kg. The BDE-209 concentration of the system containing the TiO_2_-Yi-Li immobilized chitosan beads had decreased to 13.81 mg/kg on D42 and to 8.22 mg/kg on D70. The remaining ratio of BDE-209 was calculated for this system to be 0.41 after 70 days of degradation. The BDE-209 concentration of the system containing the Yi-Li immobilized chitosan beads was found to have decreased to 14.93 mg/kg on D42 and to 10.69 mg/kg on D70. The remaining ratio of BDE-209 was calculated to be 0.53.

Debromination of BDE-209 has been reported to occur by biodegradation and by photolysis. The amount of bromide released into the clay soil slurry was measured for both systems in order to assess the debromination of BDE-209. The TiO_2_-Yi-Li immobilized chitosan beads with UVA irradiation showed a higher BDE-209 degradation ability than adding the beads containing Yi-Li bacteria alone. The TiO_2_-Yi-Li immobilized chitosan beads with 365 nm UVA irradiation produced bromide concentrations that increased from 0.64 mg/L on D0 to 27.69 mg/L on D70. On the other hand, the Yi-Li immobilized chitosan beads with UVA irradiation produce bromide concentrations that increased from 1.19 mg/L on D0 to 22.95 mg/L on D70.

#### 3.2.2. Analysis of the Bacterial Number Present during the TiO_2_-BDE209 Degradation Microorganisms

**Figure 7:**
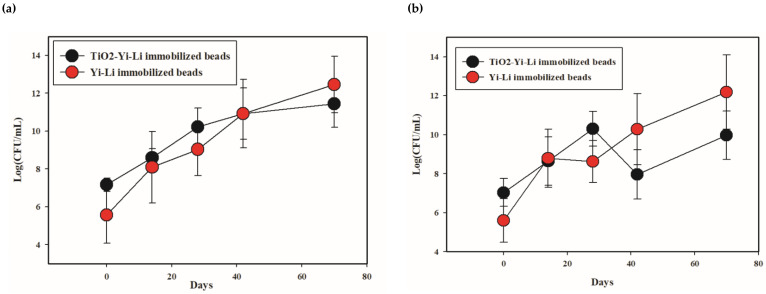
The number of bacteria present during BDE-209 degradation: (**a**) heterotrophic bacteria; (**b**) BDE-209-degrading bacteria. The Y axis is a log scale.

Changes in bacterial populations over time were estimated by plating samples on R2A agar and BDE-209-MSA agar containing BDE-209 as a sole carbon source; samples obtained during the UV irradiation-biodegradation process with TiO_2_-Yi-Li immobilized chitosan beads and with Yi-Li immobilized chitosan beads during the biodegradation process are compared in Figure 7.

The average number of bacteria present increased over time during the biodegradation process with Yi-Li immobilized chitosan beads. The total number of bacteria present growing on R2A agar increased from 3.67 × 10^5^ CFU/g on D0 to a high of 2.88 × 10^13^ CFU/g on D70. A similar trend was found for the number of bacteria present growing on BDE-209-MSA-agar, which increased from 4 × 10^5^ CFU/g on D0 to 1.56 × 10^13^ CFU/g on D70. Furthermore, the average number of bacteria present also increased during the UV irradiation-biodegradation process with TiO_2_-Yi-Li immobilized chitosan beads. The total number of bacteria growth on R2A agar present during the UV irradiation-biodegradation process with TiO_2_-Yi-Li immobilized chitosan beads increased from 1.45 × 10^7^ CFU/g on D0 to 2.72 × 10^12^ CFU/g on D70. The number of bacteria present growing on BDE-209-MSB agar increased from 1.08 × 10^7^ CFU/g on D0 to 9.27 × 10^10^ CFU/g on D70. However, the number of bacteria present showed a decrease to 9.26 × 10^9^ CFU/mL on D42. From this it is inferred that the number of Yi-Li bacteria present was affected by the continuous UV irradiation present in the latter system, although it was able to recover.

#### 3.2.3. Bacterial Community Analysis of the BDE-209-Degrading Microorganisms

**Figure 8:**
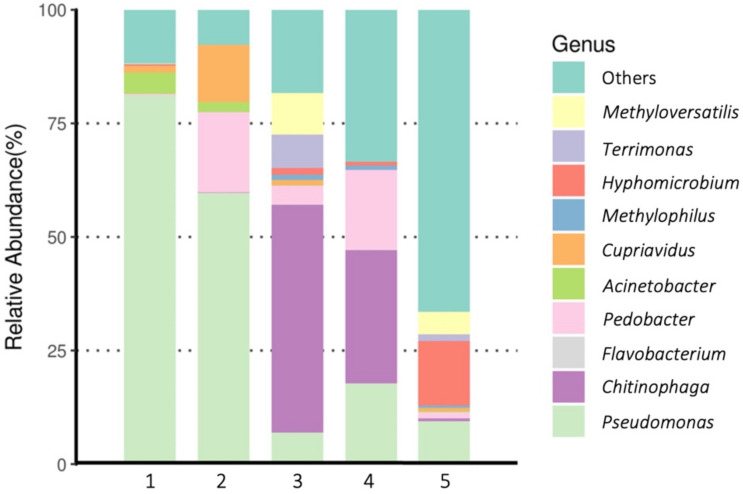
Dominant genus-level microorganisms presented in the BDE-209 contained a clay-slurry system during the degradation by the TiO_2_-Yi-Li immobilized chitosan beads coupled with UVA irradiation. Value on the X axis is defined as 1: D0, TiO_2_-Yi-Li immobilized beads; 2: D0, Yi-Li immobilized beads; 3: D56, TiO_2_-Yi-Li immobilized beads; 4: D56, Yi-Li immobilized beads; 5: Inoculum of Yi-Li bacterial mixed cultures (control).

Figure 8 shows the “top 10” genus-level analysis of the bacterial communities present in the clay slurry microcosm on D0 and the 56th day (D56) during the BDE-209 degradation experiment. On D0, the dominant bacteria were *Pseudomonas* spp. and *Acinetobacter* spp. for both bead systems. 

On D56, when the community structure of the TiO_2_-Yi-Li immobilized chitosan beads with UVA group system was explored, the dominant bacteria were now *Chitinophaga* spp. (50.17%), *Methyloversatilis* spp. (9.11%), *Terrimonas* spp. (7.41%) and *Pseudomonas* spp. (6.96%). This should be compared with the D56 of the Yi-Li immobilized chitosan beads with UVA system, where the dominant bacteria were *Chitinophaga* spp. (29.33%), *Pseudomonas* spp. (17.76%), *Pedobacter* spp. (17.50%), *Cephaloticoccus* spp. (3.46%) and *Isosphaera* spp. (3.40%).

## 4. Discussions

### 4.1. Advantages of TiO_2_-Yi-Li Immobilized Chitosan Beads When Carrying out BDE-209 Degradation

The biodegradation kinetics of BDE-209 in a clay/water system can be described as first-order. The first-order rate constants (k) for BDE-209 degradation containing TiO_2_-Yi-Li immobilized chitosan beads in the present study decreased in the order TiO_2_-Yi-Li immobilized chitosan beads (k = 0.166, 1/day) > Yi-Li immobilized chitosan beads (k = 0.132, 1/day). Previously, the rates of BDE-209 degradation in a clay slurry system have been ranked for an aerobic novel bioslurry reactor in the order: coupled UV photolysis-biodegradation (k = 0.0131, 1/day) > UV photolysis alone (k = 0.011, 1/day) > biodegradation alone (k = 0.01, 1/day) [10]. The slowest rate constant for BDE-209 degradation was found to be 0.0054 1/day when the Yi-Li bacterial-mixed culture was added, and the degradation carried out in the dark [23].

Biodegradation is not the only mechanism to remove BDE-209 in a clay/water system between adding TiO_2_-Yi-Li immobilized beads and Yi-Li-immobilized beads. The TiO_2_-Yi-Li immobilized beads contain, at day 0, more viable CFUs as compared to Yi-Li-immobilized beads (at least two orders of magnitude). However, bacterial number of TiO_2_-Yi-Li immobilized beads is restricted by both photolysis and photocatalysis after 28 h during BDE-209 degradation. It is inferred that the better degradation performances of BDE-209 by adding TiO_2_-Yi-Li immobilized beads is measured, considering the synergistic effect of TiO_2_ particles and the metabolic potential of bacteria entrapped in the beads.

There are some advantages to the use of immobilized bacterial cells for soil bioremediation, biodegradation, and biotransformation of ECs in wastewater treatment plants. First, compared with clay slurries systems utilizing suspended microorganisms, immobilized bacterial beads offer good biocompatibility, good bioavailability, a low cost and ease of preparation. Secondly, the suspended TiO_2_ powders provide a significant level of photocatalytic activity compared to other immobilized cells techniques [31]. The use of TiO_2_ nanoparticles not enclosed in beads is likely to result in widespread distribution of these nanoparticles into the environment, and this could become a significant secondary contamination problem. Finally, TiO_2_-Yi-Li immobilized chitosan beads are able to entrap both suspended nano-TiO_2_ particles and significant numbers of BDE-209 biodegrading bacteria with a higher metabolic activity; both of which can carry out excellent removal of BDE-209 in a clay slurry system. Multiple mechanisms, namely UV photolysis, photocatalysis, and biodegradation, are expected to occur at the same time during the TiO_2_-Yi-Li immobilized chitosan bead remediation process. The TiO_2_ immobilized chitosan beads technique would seem to be one of the best practical methods for remediating ECs ex-situ without causing significant environmental contamination.

### 4.2. Evaluation of the Optimal Immobilization Matrix for TiO_2_-Microbes Immobilized Beads to Be Used in an Aerobic Clay Slurry Microcosm

Alginate and chitosan have been shown to have good compatibility when used an immobilization matrix [32]. Since the entrapment immobilization process results in an intermediate binding force and a high stability, it seems likely that the microbes will be able to survive during the immobilization process. Immobilization in alginate beads has been reported to be a gentler and better method of microbial immobilization [32]. However, the mechanical strength of alginate beads is relatively weaker than that of chitosan beads [33]. The TiO_2_ immobilized chitosan beads preparation protocol was developed and modified on the basis of several studies [26,27,28]. In order to immobilize TiO_2_ particles and microbes together in beads and use these to treat ECs in a clay slurry system, the beads need to be relatively strong in terms of mechanical strength; this is because they must maintain their shape in a stirred clay slurry system. Additionally, the immobilized beads need enough mechanical strength to allow them to be potentially used in aerobic bioslurry reactors for ex-situ bioremediation when future applications are developed [34]. Thus, chitosan immobilization with bead-surface reinforcement by glutaraldehyde treatment was selected as the immobilization method to be used in this study.

**Figure 9:**
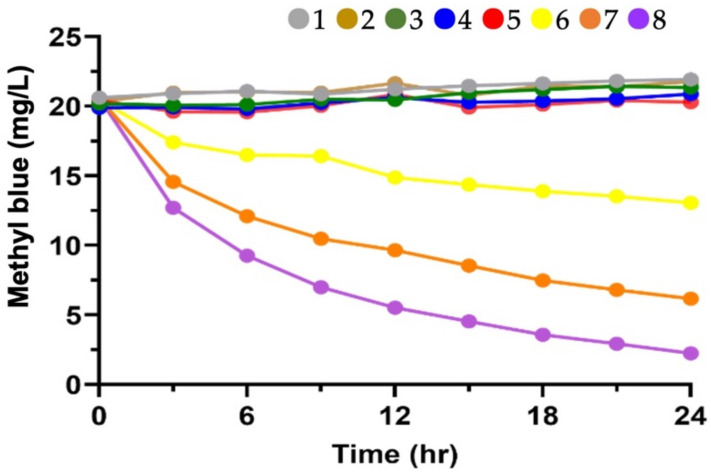
Degradation of methyl blue by TiO_2_ immobilized chitosan beads. No. of legends (●) is defined as variants of Table 4. The average value is presented here without error bars due to the very small variations in the values over the three replicates.

MB was selected as the model organic contaminant to evaluate the degradation performance of the TiO_2_ immobilized chitosan beads in this study. Figure 9 shows the TiO_2_ immobilized chitosan beads retain their photocatalytic activity in the presence of UVA irradiation throughout the experiment.

The percentage of MB degradation (%) under UV irradiation became lower and followed the order: 0.1% TiO_2_ immobilized chitosan beads (89.11%) >0.05% TiO_2_ immobilized chitosan beads (70.02%) >0.01% TiO_2_ immobilized chitosan beads (19.08%). The pseudo first-order-rate constants were calculated to be: 0.1% TiO_2_ immobilized chitosan beads (0.65, 1/h) >0.05% TiO_2_ immobilized chitosan beads (0.51, 1/h) >0.01% TiO_2_ immobilized chitosan beads (0.13, 1/h). Degradation of MB did not occur under the five control conditions, which are variants 1–5 listed in Table 2. These results indicated there was no absorption of MB onto the TiO_2_ immobilized chitosan beads over 24 h, and the degradation was brought about by TiO_2_ photocatalysis. In addition, Figure S3 shows the photograph of TiO_2_ immobilized chitosan beads before and after MB degradation. Beads have retained their shape and have shown good stability and mechanical strength while in the stirring system. They maintained their shape and diameter throughout the photocatalytic degradation experiment.

### 4.3. The Bacterial Communities Involved in the BDE-209 Biodegradation in an Aerobic Clay Slurry Microcosm

Many bacteria identified in this study are capable of biodegrading BDE-209, as well as other PBDE congeners and aromatic POPs. Both *Pseudomonas* spp. and *Acinetobacter* spp. have been reported to have the ability to degrade BDE-209 and are often found in PBDEs contaminated natural habitats [6,35]. The percentage of *Methyloversatilis* spp. and *Terrimonas* spp. was significantly higher in the TiO_2_-Yi-Li immobilized chitosan beads with UVA system compared to the system lacking TiO_2_. These two bacteria have been reported to be UV-resistant bacteria and have also been shown to have the ability to survive exposure to TiO_2_ [6,36]. In addition, the ability of *Methyloversatilis* spp. to degrade benzene has been reported, which is suggestive of the ability to break down BDE-209 [37]. Bacterial enrichment cultures that included *Cephaloticoccus* spp. have been used to biotransform mycotoxin deoxynivalenol into less toxic metabolites [38]. *Chitinophaga* spp. were present in significant numbers on the D56 in both groups. This might be attributable to the ability of *Chitinophaga* spp. to produce extracellular chitinase; this will degrade the chitin that forms the main material of the immobilized beads. Thus, *Chitinophaga* spp. would seem to be utilizing chitosan as a carbon resource rather than BDE-209, and as a result have become the dominant bacteria by D56 [39]. *Isosphaera* spp. of aerobic Planctomycetes on the D56 also are chitinolytic microorganisms that are present in wetland soils; they are known to be able to biodegrade the complex heteropolysaccharides present in EPS and cell walls; this seems to be their main ecological function [40].

Photocatalytic reactions create an environment in which some bacterial species do not thrive; this is due to conditions where there are high level of free radicals such as OH· that have been created by the TiO_2_-UVA irradiation reaction. For example, the percentage of *Pseudomonas* spp. present can be seen to be a significant difference in both systems, namely 17.76% in the Yi-Li immobilized chitosan beads that lack TiO_2_ in their UVA system compared to a lower level of 6.69% in TiO_2_-Yi-Li immobilized chitosan beads, both on D56. On the other hand, some other bacterial species are able to survive and then begin to play important roles in the TiO_2_-Yi-Li immobilized chitosan beads with UVA system, compared with the Yi-Li immobilized chitosan beads with UVA system, again both on D56. *Chitinophaga* spp. have been reported to be involved in a combination of non-thermal plasma and biological processes [41]. Non-thermal plasma involves in the acceleration of primary electrons by collision with background molecules, and this then produces highly reactive free radicals (OH and O·). *Pedobacter* spp., when exposed to a cold temperature and high UV radiation, have been shown to turn on an important antioxidant system and produce a variety of pigments that belong to the carotenoids group; these are capable of preventing oxidative damage [42].

## 5. Conclusions

This study describes the development and characterization of a novel immobilization technique whereby TiO_2_ and a BDE-209-degrading bacterial mixed culture are trapped in chitosan beads. It was found that this system is able to effectively remove BDE-209 in a clay slurry system, while at the same time decreasing the risk of secondary contamination of the environment with TiO_2_. The TiO_2_-Yi-Li immobilized chitosan beads retained both photocatalytic and biodegradation activity after the various steps needed for immobilization were carried out. Importantly, the Yi-Li microbes added to the beads ability to biodegrade an EC and, furthermore, this mixed culture was able to tolerate long-term UVA irradiation, as well as exposure to the high levels of free radical generated by the TiO_2_-UV photocatalytic reaction and the UV irradiation itself. In this system, there are three degradation mechanisms in action—microbial bioremediation, photocatalysis and UV degradation—and all of these are likely to have contributed to the breakdown of the high-brominated PBDEs present in the system; this will have produced low-brominated PBDEs congeners. Thus, the TiO_2_-Yi-Li immobilized chitosan beads are an approach with strong potential for treating ECs contaminated soils. Based on this immobilization procedure, further investigations are needed to increase the mechanical strength of immobilized beads. Such an improvement would mean that the beads could be reused, which would make the treatment of environmental pollutants even more cost effective. Furthermore, the bacteria that are able to tolerate and thrive under the multiple environmental stresses could then be isolated and used to create a new culture that can be added to a new batch of beads. Such a culture, when combined with this new immobilization technique, should have a significantly higher BDE-209 removal efficiency than the Yi-Li culture used here.

## Figures and Tables

**Table 1:** Physical-chemical characteristics of the clay used in this study ^1^.

Clay (Abbreviation)	Chemical Composition ^1^ (%)	Source	BET-(N_2_) SA(m^2^/g)	SOM (%)	CEC (meq/100)
Texas Montmorillonite STx-1	SiO_2_: 70.1, Al_2_O_3_: 16.0, TiO_2_: 0.22, Fe_2_O_3_: 0.65, FeO: 0.15, MnO: 0.009, MgO: 3.69, CaO: 1.59, Na_2_O: 0.27, K_2_O: 0.078, F: 0.084, P_2_O_5_: 0.026, S: 0.04	Gonzales County, TX, USA	83.79	0	84.4

^1^: based on the website http://www.clays.org/sourceclays_data.html (accessed on 17 January 2022).

**Table 2:** Details of the experimental conditions set up for the MB degradation experiments using different concentration of TiO_2_ immobilized chitosan beads.

Variants	Light ^1^	Beads	Type of Beads
1	UV	No	
2	Dark	No	
3	UV	Yes	chitosan beads
4	Dark	Yes	chitosan beads
5	Dark	Yes	0.05% TiO_2_ immobilized chitosan beads
6	UV	Yes	0.01% TiO_2_ immobilized chitosan beads
7	UV	Yes	0.05% TiO_2_ immobilized chitosan beads
8	UV	Yes	0.10% TiO_2_ immobilized chitosan beads

^1^ The UV light source used in this study was a 365 nm LED UVA light source set at 2–2.4 mW/cm^2^.

**Table 3:** Details of the viability assay used on the Yi-Li Bacteria, including the various chemicals used during the immobilization procedure.

Step	Chemicals	UV	Contacting Time
1	Control-0.85% NaCl	No	Immediately
2	1% acetic acid	No	Immediately
3	0.1% TiO_2_ in 1% acetic acid	No	Immediately
4	0.05% TiO_2_ in 1% acetic acid	No	Immediately
5	1% sodium tripolyphosphate	No	Immediately
6	0.25% glutaraldehyde	No	Immediately
7	1.2% chitosan dissolved in 1% acetic acid	No	30 min.
8	1.2% chitosan dissolved in 1% acetic acid	No	3 h.
9	Control-0.85% NaCl	Yes	3 min.
10	1% acetic acid	Yes	3 min.
11	Chitosan-0.1% TiO_2_ mixed suspension	Yes	3 min.
12	Chitosan-0.05% TiO_2_ mixed suspension	Yes	3 min.
13	Chitosan-0.01% TiO_2_ mixed suspension	Yes	3 min.

**Table 4:** The total weight of the regular-shaped immobilized beads manufactured using various concentration of TiO_2_-chitosan suspension and sodium tripolyphosphate.

Batch	Chitosan (*w*/*v*) ^1^	Sodium Tripolyphosphate (*w*/*v*)	Glutaraldehyde (*v*/*v*)	Weight of Immobilized Cells (g) ^2^
**1**	2.0%	3%	0.25%	NA ^3^
**2**	3.0%	3%	0.25%	NA ^3^
**3**	3.5%	3%	0.25%	NA ^3^
**4**	4.0%	3%	0.25%	4.6
**5**	4.5%	3%	0.25%	5.5
**6**	1.2%	1%	0.25%	12.5

^1^: Chitosan dissolved in 1% acetic acid, ^2^: Generated, ^3^: Not available for regular-shaped immobilized beads.

## Supplementary Materials

The following are available online at https://www.mdpi.com/article/10.3390/microorganisms10020402/s1, Figure S1. Schematic of the TiO_2_-immobilization procedure., Figure S2. The viability of the Da-An bacteria in the various chemicals used in the immobilization procedure. Value 1–6 on the X axis is defined as each step of Table 3, including the various chemicals used during the immobilization procedure. Step: 1: Control-0.85% NaCl; step 2: 1% acetic acid; step 3: 0.1% TiO_2_ in 1% acetic acid; step 4: 0.05% TiO_2_ in 1% acetic acid; step 5: 1% sodium triphosphate; step 6: 0.25% glutaraldehyde. Figure S3. Photographs of the TiO_2_ immobilized chitosan beads. (a) Before use in the dye degradation experiment. (b) After use in the dye degradation experiment.
