# Supplementary material for: Degradation of Decabromodiphenyl Ether in an Aerobic Clay Slurry Microcosm Using a Novel Immobilization Technique
Source: Microorganisms. 2022 Feb 9;10(2):402. doi: 10.3390/microorganisms10020402 (PMC8877889; doi:10.3390/microorganisms10020402)
Supplement: Supplementary file 1 [file microorganisms-10-00402-s001.zip › microorganisms-1582578- supplementary.pdf]

Supplementary Materials

Degradation of Decabromodiphenyl Ether in an Aerobic Clay Slurry Microcosm Using a Novel Immobilization Technique

Jung-Shan Hsu [1,2], Ting-Yu Yu [1], Da-Jiun Wei [1], Wann-Neng Jane [3], Yi-Tang Chang [1,*]

[*]Corresponding author:

Yi-Tang Chang, Ph.D.

Distinguished Professor, Department of Microbiology

Dean, School of Science

Soochow University,

70, LinXi Rd., Shinlin District, 11102, Taipei, Taiwan

E-mail: ytchang@scu.edu.tw; timchang08@gmail.com

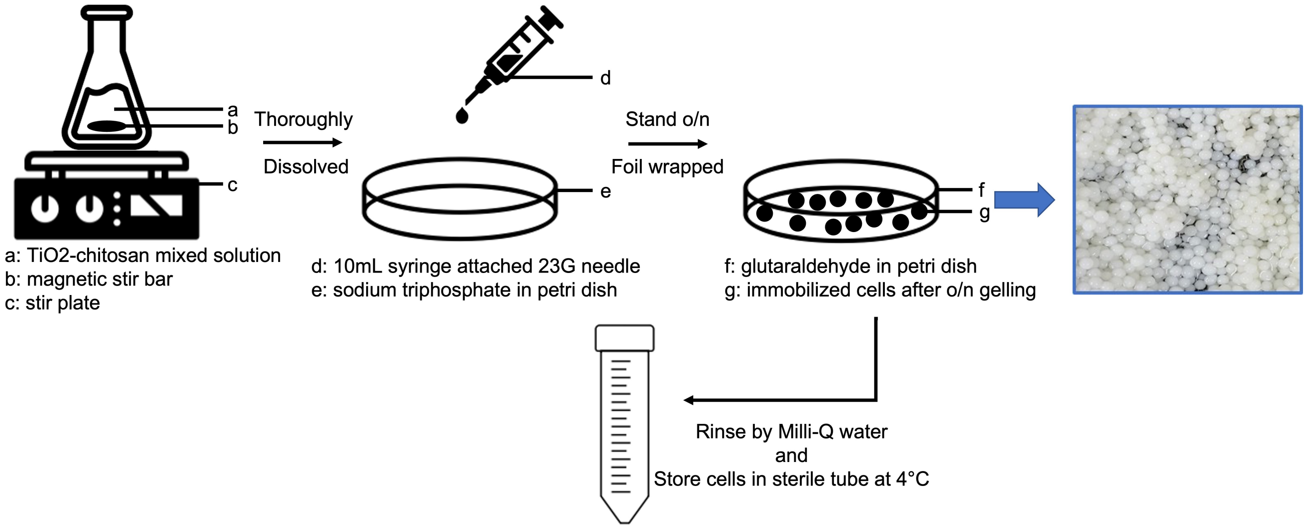

Figure S1. Schematic of the TiO_2-immobilization procedure.

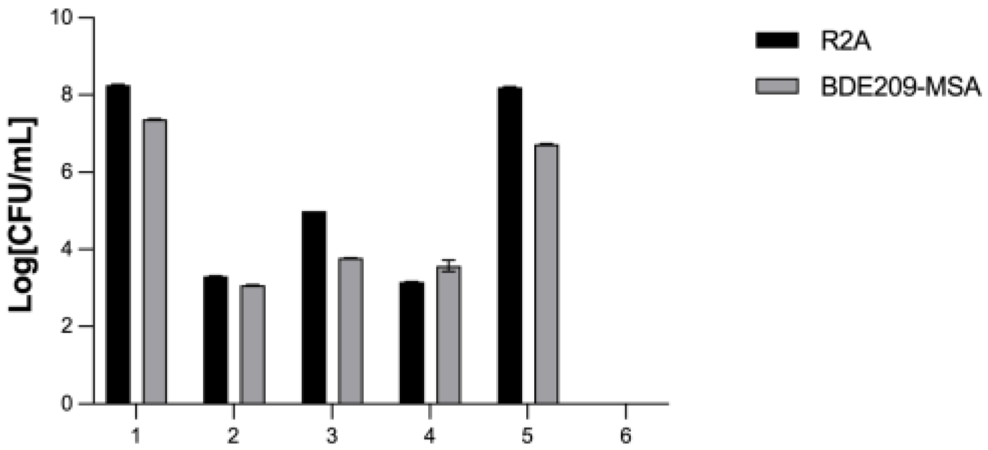

Figure S2 The viability of the Da-An bacteria in the various chemicals used in the immobilization procedure. Value 1-6 on the X axis is defined as each step of Table 3, including the various chemicals used during the immobilization procedure. Step: 1: Control-0.85% NaCl; step 2: 1% acetic acid; step 3: 0.1% TiO_2 in 1% acetic acid; step 4: 0.05% TiO_2 in 1% acetic acid; step 5: 1% sodium triphosphate; step 6: 0.25% glutaraldehyde.

(**a**) (**b**)

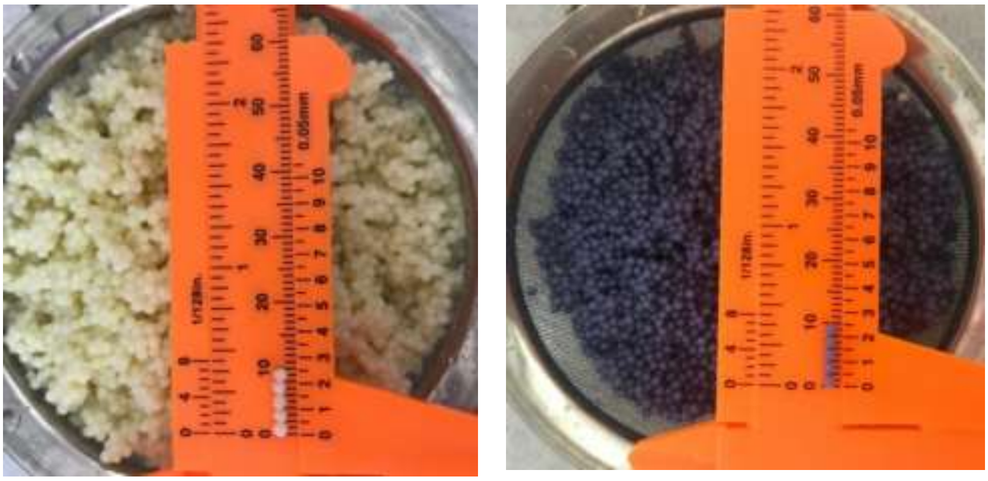

Figure S3. Photographs of the TiO_2 immobilized chitosan beads. (**a**) Before use in the dye degradation experiment. (**b**) After use in the dye degradation experiment.
